# Spectator attendance without spectators: stakeholder insights from a national athletics championships

**DOI:** 10.3389/fspor.2026.1818383

**Published:** 2026-04-15

**Authors:** Monika Písečná, Iveta Cihová

**Affiliations:** Department of Track and Field and Sport Conditioning, Faculty of Physical Education and Sports, Comenius University Bratislava, Bratislava, Slovakia

**Keywords:** athletics, attendance, fan engagement, national athletics championships, spectator, stakeholder

## Abstract

**Introduction:**

Spectator attendance at the national athletics championships in Slovakia has been low for a long time, despite free admission. Not even the elevated performance of Slovak athletes in certain disciplines and the growing media interest in leading Slovak athletes has contributed to higher spectator attendance at these events. For this reason, the aim of this study was to map the current state of attendance, to identify satisfaction with selected factors that condition low spectator attendance and to develop recommendations for the Slovak Athletics Federation.

**Methods:**

The study employed cross-sectional ex post facto research, which was based on questionnaire data collected during the Slovak Athletics Championships. The Motivation Scale for Sport Consumption questionnaire, which is focused on surveying basic data about the respondents, their motivation for attending an athletics event, their satisfaction with the championships with regard to selected aspects of the event and their motivation to consume sport, was used. The research sample comprised 39 respondents from among athletes, coaches, family members and friends. The Chi-square goodness of fit test and Mann-Whitney U-test were used to analyse the data.

**Results:**

The results indicated that all respondents had a direct personal or professional connection to athletics, and independent spectators were not represented in the set. Through the questionnaire, we determined that the accompanying programme of the championships had a statistically significant negative impact on the satisfaction of stakeholders (*p* = 0.003). In contrast, the positive satisfaction of stakeholders with the date and venue of the championships was statistically significant (*p* = 0.003). Direct participants expressed statistically higher satisfaction compared to indirect participants in the areas of event organisation (*p* = 0.004; *r* = 0.46) and transportation and parking (*p* = 0.039; *r* = 0.33).

**Discussion:**

Direct and indirect participants have a similar psychological profile, since they are motivated by the same areas, particularly the physical attractiveness of the athletes and the drama of the competition. The low attendance at Slovak Athletics Championships is a result of the event's limited attractiveness to the wider public and its poor promotion. The audience is comprised exclusively of people with a direct relationship to athletics, which suggests the absence of effective strategies aimed at reaching potential spectators. What's more, the results of the study indicate that the sporting experience itself is not enough to create a comprehensive spectator experience and highlight the need for appropriate promotional activities. On the basis of these results, recommendations were developed for the Slovak Athletics Federation.

## Introduction

Spectator attendance is widely regarded as an important indicator of the social and economic relevance of sport events. However, many sport organizations, particularly those operating in smaller markets or within less commercially visible sports, face persistent challenges in attracting spectators to live competitions. Previous research indicates that spectators' attendance decisions are influenced not only by the quality of the sporting performance but also by various aspects of the event environment, such as accessibility, stadium services, and the overall atmosphere of the venue (1). Despite these challenges, sport continues to provide a range of benefits for different stakeholder groups, including athletes, coaches, family members and friends.

Sport and sporting events, as such, offer many benefits, though these benefits vary depending on the group the participants belong to. The difference is whether you are attending a sporting event as part of your profession or because it is a hobby and entertainment for you (2). Being aware of this fundamental difference is the first step towards acknowledging that sports audiences are not homogeneous, which has a huge impact on sports managers and marketers (3). If you are a spectator, the main benefit of sport for you is the emotional experience. Sporting events evoke emotions in both spectators and participants, and this creates a strong bond with the event (4) and makes them perceive participation in the event as an escape from everyday life (5). Many spectators, whether direct or indirect, attend and watch sporting events for the emotions and experiences that sport evokes in them, and these emotional benefits are a key factor in sports consumption and fan loyalty. A family member or friend who comes to a sporting event to cheer on a specific athlete—i.e., they do not go to events to consume sport as such—has a greater the experience of a sporting event. In Slovakia, we have many such passionate family members and friends, and without them the stands would be empty. There are also fans who take part in sporting events as a leisure activity and not as participation in a prestigious sporting competition (6).

For Slovak athletes, the benefits of sport can mean that it creates a job for them, and they are thus able to make a living doing sport. The successes and media interest of professional Slovak athletes also have a positive impact on obtaining sponsors and the number of them obtained. A smaller portion of Slovak athletes are included in ministerial sports centres, which help finance and cover their preparation for the season. We have three ministerial sports centres in Slovakia that are financed by three different ministries: the National Sports Centre (Ministry of Tourism and Sports) (7), the Military Sports Centre Dukla Banská Bystrica (Ministry of Defence) (8) and the Police Sports Centre (Ministry of the Interior) (9). Another source of income for Slovak athletes comes from the Slovak Athletics Federation and, in the case of a high level of performance and success, they also receive funding from the Slovak Olympic and Sports Committee. However, only a very small percentage of Slovak athletes can truly make a living just by being a professional athlete; this is one of the main reasons why the largest number of athletes in Slovakia end their careers in the junior category.

Sport is at the same time a strong motivator in the area of health and physical activity. One study in Japan examined the relationship between attendance at sporting events and the subjective perception of one's own health among adult Japanese residents over the course of 12 years. The results of the analysis show that direct spectators of sporting events are 33% more likely to be in better health than spectators who do not attend such events in person (10). Passively spending leisure time at sporting events builds social and emotional needs that protect spectators from the negative effects of stress (10). Performance and top-level sports events significantly increase attendees' life satisfaction and build a positive image of the destination (11). We also place benefits of an economic, social, political, environmental nature, along with individual health, personal development, inspiration and role models among the benefits of sport (12).

Athletics is considered a non-mainstream sport in Slovakia, as more attention is paid to team sports such as football and hockey. National athletics events in Slovakia have low attendance despite offering free entry. Therefore, in the study, we address participants' satisfaction with selected factors, their motivation and demotivation to come to athletics events and questions related to identification with given statements using the standardised Motivation Scale for Sport Consumption questionnaire (13). The factors we examine are event organisation, event commentary, refreshment options, transportation and parking, the overall atmosphere, quality of infrastructure, date and venue of the event and the accompanying event programme.

The process of marketing communication is highly demanding and should be handled by a marketing specialist, who creates a marketing plan and budget in advance (14). Marketing specialists manage all marketing activities, from the preparation to the distribution and subsequent implementation of promotional materials, as well as to the selection of appropriate communication channels for individual marketing activities (15). A sporting event is managed by a marketing strategy, but in reality this does not always work that way, as smaller sporting events and national sports federations have a smaller number of employees who perform multiple jobs and tasks (16). As a result, a sporting event is often undersized in terms of marketing. For successful marketing of a sporting event, it is necessary to divide the audience by place of residence, as this factor significantly affects the willingness to attend the event again, but it is also important to pay attention to the age, previous experience with sports (17) and gender of participants (18). Higher marketing success can be ensured by the use of personalised marketing and digital platforms to strengthen the emotional bond with the audience (19, 20).

Among the other factors that draw spectators to sporting events are the desire for entertainment, admiration for the sport and affection for specific athletes; on the other hand, the timing and price of tickets can negatively affect the level of attendance (21). At present, the trend is to organise accompanying events alongside sporting events. Such accompanying events can have a cultural and educational nature and be intended for the general public (22).

The authors also include factors such as aesthetics, interaction, success, skills, knowledge, performance and drama (17) among the key motivators of participation in a sporting event. For a broader representation of the factors that affect attendance at sporting events, we present the following 10 items: identification, player, community, family, entertainment, drama, escape, skill, achievement and the social side (23). In a study examining psychological and social factors that influence attendance at matches in Japan, it was found that team identification, escape from reality and social interaction were the main influential factors (23). From the perspective of young people in Slovenia and Bosnia and Herzegovina, cultural background, national identity and gender were among the major reasons why young people attend sporting events or follow them in the media (24).

A direct correlation was found between the quality of the environment and fans' willingness to return to matches, which was influenced by cleanliness, the availability of parking and the level of service (25). The link between the sporting experience and appropriate infrastructure is likewise a factor for higher attendance at sporting events (26).

Low attendance at sporting events occurs for several reasons. The results of the study showed that the tendency of spectators to take part was directly affected by their level of satisfaction, and this satisfaction showed a significant correlation with the quality of the game, the physical environment and the quality of the result (27). A study was conducted in Egypt that focused on low attendance at sporting events in individual sports. It showed that insufficient media coverage, poor promotion by national sports federations and the cultural dominance of football were the reasons for the low attendance (28). The mere existence of sport is not sufficient to increase public interest in watching sport; it is necessary to focus on the internal psychological factors of the viewer and how they perceive and have an emotional relationship with the sport (29). The absence of sports personalities and the weaker results of the national team compared to the world's top teams may also affect the lower attendance, but more intensive promotion and community building through schools can help make matches more attractive (30). If we make social media promotion more effective, not only will stable supporters attend sporting events, but also occasional fans and younger generations who make decisions mainly based on digital engagement (31). A study carried out in Qatar stressed that social media, particularly Facebook, significantly strengthen spectators' intentions to attend an event in person (21).

Email marketing has for many years been a popular form of communication with customers and is also used for many purposes from a marketing perspective (32). The most suitable alternative for the Slovak Athletics Federation is email marketing through newsletters. This is a form of mass email that is distributed to registered subscribers. Its most common task is to inform customers about news and sometimes about organised events (32). The newsletter should not give subscribers the impression that it is spam; otherwise there is a high probability that the subscriber will unsubscribe from the newsletter. Therefore, individual newsletters should be sent sparingly and should contain unique information for the subscriber.

### Research gap

Despite extensive research into audience motivation and attendance at sporting events, most studies have focused primarily on professional leagues and major sporting events (33). Much less attention has been paid to less popular sports and sporting events, where organizers often have limited marketing resources and low attendance. In addition, previous studies have focused primarily on the motivation of spectators from the general public rather than stakeholders involved in the sporting event (athletes and coaches) or indirect participants who had close ties to direct participants (family members and friends). Understanding these perspectives can provide valuable information on how organizers can increase spectator attendance at their events.

Research question 1: How satisfied are stakeholders with selected organizational aspects of the Slovak Athletics Championships?

Research question 2: Do direct participants (athletes and coaches) and indirect participants (family members and friends) differ in their level of satisfaction with selected event factors?

## Materials and methods

### Research design

This research has the character of a cross-sectional ex post facto study, whose results are obtained on the basis of a single measurement at time t0 on the research set (34). The research is based on questionnaire data that was collected during the Slovak Athletics Championships on 2–3 August 2025 in Banská Bystrica. The schedule of the Slovak Athletics Championships was on the first day from 2:55 p.m. to 7:00 p.m. and on the second day from 10:00 a.m. to 2:45 p.m (35).

### Participants

A convenience sampling method was used and a total of 39 respondents took part in the study by filling out the questionnaire during the Slovak Athletics Championships. The research sample consisted of 66.66% women and 33.34% men. The average age of all respondents was 31.28 years (standard deviation = 13.44); for women the average age was 31.42 years (standard deviation = 13.36), and for men the average age was 31.00 years (standard deviation = 14.15).

From the viewpoint of their role at the event, the largest representation of respondents was athletes 53.85% (*n* = 21), then family members 20.51% (*n* = 8), coaches 15.38% (*n* = 6) and friends 10.26% (*n* = 4). The structure of respondents thus reflects a combination of direct participants in the sports environment (athletes and coaches) and the indirect participants (family members and friends). Nearly 70% of respondents said that they take part in more than five athletic events during the year. In most cases these are the Slovak Indoor and Outdoor Athletics Championships, the Athletic League, the Tipos P-T-S meeting, school athletics competitions and district and regional athletics competitions.

### Data collection

Data collection was done via an online questionnaire that was distributed during the Slovak Athletics Championships by means of a QR code throughout the stadium. An online questionnaire offers efficiency, and a large number of questionnaires can be distributed simultaneously (36). Respondents filled in the questionnaire anonymously through their own mobile devices and in case respondents were not familiar with using their mobile devices, we had printed versions of the questionnaires ready. This method of data collection enabled a quick and efficient approach to the event participants. The questionnaire was distributed in the Slovak language, and initially all respondents received uniform information about the questionnaire. This included information about the name of the research, the aim of the research, the target group of respondents, the time to complete the questionnaire, funding from NextGenerationEU sources and that by completing the questionnaire, respondents give their consent to the processing of data for research purposes. The questionnaire was divided into four sections. The first section focused on collecting basic data about the respondent. The second investigated the respondent's motivation for coming to the athletics event. The third section dealt with satisfaction with the athletics event, and the fourth section focused on obtaining data from the standardised Motivation Scale for Sport Consumption questionnaire (13). Most questions were assessed using a Likert scale, which allowed us to make quantitative comparisons between groups. In addition, we used open-ended questions in the questionnaire, marking one of the selected options, as well as marking multiple options. Participation in the research was voluntary and anonymous. Prior to completing the questionnaire, respondents were informed about the instructions, purpose of the research and that their answers would be used for research purposes. The data collection and processing did not contain any personal identifiers.

### Data analysis

The data acquired from the online questionnaire were analysed on two levels. In the first part, we assessed the participants' satisfaction with eight factors of the event using a 5-point Likert scale (1 = very dissatisfied, 5 = very satisfied). To meet the assumptions of the Chi-Square Goodness-of-Fit test, the original five-point Likert scale was reduced to three categories (satisfied, neutral, dissatisfied). Although this approach reduces data variability and may affect the interpretation of the results, it was adopted due to the relatively small sample size (*n* = 39) and the occurrence of zero observed frequencies in some categories. Collapsing response categories is a common approach in studies with small samples, as it allows meeting the assumptions of categorical statistical tests while preserving interpretability of the data. The three-category structure enabled researchers to retain the general evaluative direction of responses (positive, neutral, negative) and ensured that the statistical analysis remained methodologically appropriate.

The Chi-Square Goodness-of-Fit test was used to examine the distribution of respondents' answers across evaluation domains. The analysis aimed to determine whether the observed response frequencies (satisfied, neutral, dissatisfied) significantly deviated from a theoretically expected uniform distribution. Cohen's w was used to assess the effect size of statistically significant relationships. Effect sizes were interpreted according to Cohen's guidelines (37): 0.10 = small, 0.30 = medium, and 0.50 = large.

The next level of analysis was focused on comparing satisfaction between two groups of respondents: direct participants (athletes and coaches) and indirect participants (family members and friends). Respondents used a five-point scale to answer the questions: very dissatisfied (1), dissatisfied (2), neither satisfied nor dissatisfied (3), satisfied (4), very satisfied (5). The Mann–Whitney *U-*test was used to identify statistically significant differences in the ratings of eight organisational factors between these groups. Given the nature of the data, the differences in responses were interpreted using the median (Mdn), first quartile (Q1), third quartile (Q3) and interquartile range (IQR). the substantive significance was determined using the r coefficient.

In the third part, the standardised Motivation Scale for Sport Consumption (MSSC) questionnaire was used. Respondents rated statements on a seven-point scale: strongly disagree (1), disagree (2), slightly disagree (3), don't know (4), slightly agree (5), agree (6), and strongly agree (7). The score for the seven main domains was calculated as the arithmetic mean of the corresponding three items for each area. In this case, we compared the agreement or disagreement of direct and indirect stakeholders with the statements belonging to individual domains. We performed the data analysis using the Mann–Whitney *U-*test and significance using the r coefficient. When interpreting ordinal data, the median (Mdn), first quartile (Q1), third quartile (Q3) and interquartile range (IQR) were used.

The assumption of normality was tested using the Shapiro–Wilk test. As the empirical data did not meet the normality assumption, non-parametric descriptive statistics were used, including the median (Mdn), first quartile (Q1), third quartile (Q3), and interquartile range (IQR). Differences between direct participants (athletes and coaches) and indirect participants (family members and friends) were examined using the non-parametric Mann–Whitney *U-*test. The effect size (r) was calculated to assess the practical significance of the observed differences. The interpretation of effect size followed Cohen's guidelines (37), 0.10 = small, 0.30 = medium and 0.50 = large.

### Data sources

Media coverage was determined based on articles published on the official website of the Slovak Athletics Federation. These were divided into the pre-event, during-event and post-event periods. In total, four articles that were published on the official website of the Slovak Athletics Federation were identified. Readership of the articles was evaluated based on the number of views of the articles publicly shown on the official website of the Slovak Athletics Federation at the time of data collection on 7 February 2026. These metrics were used descriptively only and were not interpreted as indicators of unique audience, reach or engagement.

The first article published in connection with the Slovak Athletics Championships was published shortly before the event, on 31 July 2025, and its readership was 1906 views (38). The second article was published prior to the event on 1 August 2025, and its readership was 518 reads (39). The third article was published on 2 August 2026 after the first day of the Slovak Athletics Championships as a summary of the results, and the fourth article was published after the second day of the Slovak Athletics Championships as a summary of the second day of the results. The readership of the third article was 2064 views and the fourth had 2061 views (40, 41).

The Slovak Athletics Federation used official accounts on Facebook and Instagram for media coverage of the Slovak Athletics Championships for men and women. In total, 16 posts were published on Facebook and 17 on Instagram. Stories on these two social media were excluded from the analysis due to the lack of access to the archive, which would have made the research impossible to replicate. The lower visibility of the Slovak Athletics Championships was due primarily to other athletic events that took place before and after the Slovak Athletics Championships. The Slovak Athletics Federation reported in particular on the performances of Slovak athletes at the European U23 Championships, the FISU Summer World University Games, the Summer European Youth Olympic Festival and the European Junior Championships. The event that got the most attention was the domestic international athletics event, the P-T-S meeting, which took place during the entire week after the Slovak Athletics Championships (8 August 2025).

## Results

The Chi-Square Goodness of Fit test was used to assess the distribution of respondents' answers in individual areas of evaluation of the Slovak Athletics Championships. The aim of the analysis was to verify whether the observed distribution of respondents' answers (satisfaction, neutral attitude, dissatisfaction) differs statistically significantly from the theoretically assumed uniform distribution. A statistically significant deviation from the normal distribution was identified in two areas: date and venue of the championships and accompanying event programme of the championships (Table 1). No statistically significant relationships were confirmed between the groups in the other studied factors.

**Table 1:** Differences in satisfaction with selected attendance-related factors at the Slovak athletics championships.

Area of evaluation		*n*	%	*p*	w
Event organisation	Satisfaction	19	48.7	0.116	0.33
	Neutral	9	23.1		
	Dissatisfaction	11	28.2		
Event commentary	Satisfaction	9	23.1	0.199	0.29
	Neutral	12	30.8		
	Dissatisfaction	18	46.1		
Refreshment options	Satisfaction	9	23.1	0.116	0.33
	Neutral	11	28.2		
	Dissatisfaction	19	48.7		
Transport and parking	Satisfaction	17	43.6	0.368	0.23
	Neutral	12	30.8		
	Dissatisfaction	10	25.6		
The overall atmosphere	Satisfaction	8	20.5	0.092	0.35
	Neutral	12	30.8		
	Dissatisfaction	19	48.7		
Quality of infrastructure	Satisfaction	15	38.5	0.232	0.27
	Neutral	8	20.5		
	Dissatisfaction	16	41.0		
Date and venue of the event	Satisfaction	23	59.0	**0**.003**	0.54
	Neutral	8	20.5		
	Dissatisfaction	8	20.5		
Accompanying event programme	Satisfaction	7	17.9	**0**.003**	0.55
	Neutral	9	23.1		
	Dissatisfaction	23	59.0^1^		

*n*, quantity, values in the shape quantity, frequency (%); *p*, *p* value for Chi-Square Goodness of Fit test; *w*, Cohen's w value (measure of effect size for the Chi-Square Goodness of Fit test).

** Bold values indicate statistical significance at *p* ≤ 0.01.

When evaluating the date and venue of the championships (Х^2^ = 11.54; df = 2; *p* = 0.003), the distribution of responses differed significantly from a normal distribution. From a practical point of view, this was a large effect (*w* = 0.54). Most respondents (59.0%) expressed satisfaction, indicating a favorable perception of this organizational dimension. The results showed that the date and venue of the event had a significantly positive impact on stakeholder satisfaction. Although a positive evaluation predominated, the statistical significance indicates that this satisfaction was not evenly distributed among all the groups of respondents.

A similarly statistically significant difference was recorded in the evaluation of the championship's accompanying event program (autograph sessions, children's competitions, fan zones) (Х^2^ = 11.69; df = 2; *p* = 0.003) with a large practical effect (*w* = 0.55). In this case, dissatisfaction prevailed (59.0% of respondents), which indicates a significantly negative assessment of this area. The result signals a potential deficit in the representation, quality, or attractiveness of accompanying activities in the perception of the event among individual groups of stakeholders.

In the other areas evaluated no statistically significant deviation from normal distribution was found. Although the descriptive analysis indicates certain differences in the percentage representation of individual responses, these differences did not reach the level of statistical significance and therefore it is not possible to conclude that a particular evaluation category was dominant among respondents. Specifically, these were: event organisation (*p* = 0.116; *w* = 0.33), event commentary (*p* = 0.199; *w* = 0.29), refreshment options (*p* = 0.116; *w* = 0.33), transport and parking (*p* = 0.368; *w* = 0.23), the overall atmosphere (*p* = 0.092; *w* = 0.35) and quality of infrastructure (*p* = 0.232; *w* = 0.27). The relatively high levels of dissatisfaction were recorded for the refreshment options (48.7%), the overall atmosphere (48.7%) and the event commentary (46.1%). A total of 41.0% of respondents expressed dissatisfaction with the quality of infrastructure. In contrast, transport and parking were assessed more favourably (43.6% satisfied; 25.6% dissatisfied). In summary, it can be said that the logistical and organisational aspects of the event were perceived relatively consistently among the individual groups. On the other hand, the experiential and accompanying elements of the event, particularly the accompanying event programme, showed a higher level of dissatisfaction as well as a more significant differentiation between the stakeholder groups. The identified strong effect sizes for two statistically significant factors suggest that these areas represent practically significant dimensions of the event evaluation.

The results of the comparative analysis of the Mann–Whitney *U-*test (Table 2) revealed statistically significant differences in satisfaction between direct participants (athletes and coaches) and indirect participants (family members and friends) in two areas. We found the statistically most significant differences in the event organisation (*p* = 0.004; *r* = 0.46), where direct participants evaluated satisfaction with the event positively (Mdn 4) and indirect participants, on the contrary, rated the organisation negatively (Mdn 2). Statistically significant differences also appeared in relation to transportation and parking (*p* = 0.039; *r* = 0.33), with direct participants again being more satisfied (Mdn 4) compared to indirect participants (Mdn 2.5). For other factors, such as refreshment options, accompanying event program, the quality of infrastructure, the date and venue of the event, the event commentary and the overall atmosphere, no statistically significant differences in satisfaction between direct and indirect participants of the Slovak Athletics Championships were recorded. The data obtained confirm that direct participants are more satisfied with the Slovak Athletics Championships compared to indirect participants.

**Table 2:** Differences in satisfaction with selected attendance-related factors between direct participants and indirect participants at the Slovak athletics championships.

Area of evaluation		Mdn	Q1	Q3	IQR	*p*	r
Event organisation	Direct participants	4	3	4	1	**0**.004**	0.46
	Indirect participants	2	2	3	1		
Event commentary	Direct participants	3	2	3	1	0.358	0.15
	Indirect participants	2	2	3.5	1.5		
Refreshment options	Direct participants	3	2	3.5	1.5	0.181	0.21
	Indirect participants	2	1.5	3	1.5		
Transport and parking	Direct participants	4	3	4	1	**0**.039*	0.33
	Indirect participants	2.5	1	3.5	2.5		
The overall atmosphere	Direct participants	3	2	3	1	0.729	0.06
	Indirect participants	2.5	1.5	3	1.5		
Quality of infrastructure	Direct participants	3	2	4	2	0.301	0.17
	Indirect participants	2	1.5	4	2.5		
Date and venue of the event	Direct participants	4	3	4	1	0.301	0.17
	Indirect participants	3.5	1	4	3		
Accompanying event programme	Direct participants	2	2	3	1	0.274	0.18
	Indirect participants	2	1.5	3	1.5		

Mdn, median; Q1, lower quartile (25th percentile); Q3, upper quartile (75th percentile); IQR, interquartile range; *p*, *p* value for the Mann–Whitney *U*-test; *r*, effect size for the Mann–Whitney *U*-test.

** Bold value indicates statistical significance at *p* ≤ 0.01.

* Bold value indicates statistical significance at *p* ≤ 0.05.

The third analysis was also performed using the non-parametric Mann–Whitney *U-*test. The analysis compared the opinions of direct and indirect participants regarding seven domains from the standardised Motivation Scale for Sport Consumption Motives questionnaire (Table 3), and the results did not show any statistically significant differences between the groups in any of the investigated domains. This suggests that the opinions of direct and indirect viewers were very similar. The highest evaluations were given by both direct and indirect participants in the physical attractiveness of the athletes domain, where the median reached the maximum possible value (Mdn = 7). The results were not statistically significant, however (*p* = 0.987; *r* = 0.003). In the drama domain the median was higher for direct participants (Mdn = 6.7) than for indirect participants (Mdn = 6.2), but the difference was not statistically significant (*p* = 0.256; *r* = 0.18). Both groups, however, rated this area relatively highly, which indicates a generally positive perception of the dramatic nature of the competitions. The third highest rated domain was vicarious achievement, where the median was higher for indirect participants (Mdn = 6.5) than for direct participants (Mdn = 6.3) without statistically significant differences (*p* = 0.483; *r* = 0.11). Only in the case of the assessing the vicarious achievement domain did the value of the opinions of indirect participants prevail over the value of the direct participants; in the other domains it was always the other way around. In the aesthetics domain, direct participants showed a higher median (Mdn = 6.3) than indirect participants (Mdn = 5.8), with the effect reaching a low to medium size (*r* = 0.24), but without statistical significance (*p* = 0.127). In the case of the escape domain, the medians of the groups differed only minimally (direct participants: Mdn = 6; indirect participants: Mdn = 5.7), and the difference was not statistically significant (*p* = 0.818; *r* = 0.04). The effect size indicates a negligible difference between the groups. In the social interaction domain, the median was slightly higher for direct participants (Mdn = 6) than for indirect participants (Mdn = 5.3), but the difference was not statistically significant (*p* = 0.646; *r* = 0.07). The variability of responses was moderately higher for direct participants (IQR = 2), which may indicate a wider spectrum of individual experiences. The lowest rated domain from the perspective of both direct (Mdn = 5.7) and indirect (Mdn = 5.2) participants was acquisition of knowledge, and the differences in the opinions of these two groups were not statistically significant (*p* = 0.592; *r* = 0.09).

**Table 3:** Differences in motivation scale for sport consumption motives between direct participants and indirect participants.

Area of evaluation		Mdn	Q1	Q3	IQR	*p*	*r*
Vicarious Achievement	Direct participants	6.3	6	6.3	0.3	0.483	0.11
	Indirect participants	6.5	5.8	6.8	1		
Acquisition of Knowledge	Direct participants	5.7	5	6	1	0.592	0.09
	Indirect participants	5.2	4.5	6.2	1.7		
Aesthetics	Direct participants	6.3	5.5	7	1.5	0.127	0.24
	Indirect participants	5.8	5	6.5	1.5		
Drama	Direct participants	6.7	6	7	1	0.256	0.18
	Indirect participants	6.2	6	6.7	0.7		
Escape	Direct participants	6	4.8	6.5	1.7	0.818	0.04
	Indirect participants	5.7	5	6.5	1.5		
Physical attractiveness of the athletes	Direct participants	7	6.3	7	0.7	0.987	0.003
	Indirect participants	7	6	7	1		
Social Interaction	Direct participants	6	4.7	6.7	2	0.646	0.07
	Indirect participants	5.3	4.5	6.3	1.8		

Mdn, median; Q1, lower quartile (25th percentile); Q3, upper quartile (75th percentile); IQR, interquartile range; *p*, *p* value for the Mann–Whitney *U*-test; *r*, effect size for the Mann–Whitney *U*-test.

## Discussion

The findings point to a differentiated perception of individual aspects of the championship's organization. Respondents gave a clearly positive assessment of the date and venue of the event, which can be interpreted as a correct organizational decision on the part of the organizer. On the contrary, the accompanying event program was evaluated predominantly negatively, and this trend was statistically confirmed. Including an accompanying programme at the Slovak Athletics Championships will help build a spectator base and create entertainment for participants. Regarding participants, however, it is also important to pay attention to factors that showed strong dissatisfaction among participants, though they did not show a statistically significant relationship. These factors include the event commentary, the refreshment options, the overall atmosphere and also partly the quality of infrastructure. If the goal is to increase attendance outside the circle of family members and friends, it is essential to invest in the areas with the lowest measured satisfaction, i.e., mainly in the accompanying event programme and the overall atmosphere. Based on the analysis, it can be concluded that the satisfaction of respondents was not clearly defined in all areas—in most areas, no clear preference for a specific rating was demonstrated. The most pronounced attitudes were expressed in the evaluation of the date and venue of the event and in the evaluation of the accompanying event program.

The main finding of the study is a significant difference in the perception of the quality of the event between direct participants (athletes, coaches) and indirect participants (family members and friends). This phenomenon indicates that the Slovak Athletics Championships are currently conceived mainly for athletes as a means of creating the best possible conditions for their performance, while the spectator experience is not important. The statistically most significant difference in satisfaction with the event organisation revealed that direct participants are satisfied with the organisation of the event (Mdn 4), which is not the case for indirect participants, who evaluate the event organisation negatively (Mdn 2). This state is likely caused by the fact that the organisational structure concentrates on the smooth running of the disciplines, the timetable and the technical background, which suits the athletes. A spectator, however, perceives the organisation from a different perspective, and it is evident that the needs of this group of participants also need to be addressed. We recorded similar results for transport and parking, where the dissatisfaction of indirect participants is again evident (Mdn 2.5). This is mainly caused by the fact that the car park at the athletic stadium is filled by athletes, coaches, officials and implementation teams by the time the spectators arrive, so there are only a limited number of parking spaces left for indirect spectators. The attendance of a sporting event is influenced by factors such as the overall atmosphere, spectator satisfaction, team loyalty, the possibility of personal relaxation and the influence of the team (42). If some of these factors are neglected, this is then reflected in the low spectator attendance. The satisfaction of direct participants with the given factors is further evident in the remaining factor assessments, where their satisfaction outweighs the satisfaction of indirect participants.

The absence of statistically significant differences is in this case a positive finding, as it indicates a strong agreement between direct and indirect spectators. Analysis of motivational factors through the use of the standardised Motivation Scale for Sport Consumption Motives questionnaire revealed a very similar degree of consistency between direct and indirect participants of the event. The fact that the Mann–Whitney *U-*test did not show any statistically significant differences in the seven areas examined indicates that the Slovak Athletics Championships attract participants with a very similar psychological profile. The strongest motivational factor for both groups was the physical attractiveness of athletes (Mdn = 7), which confirms that the aesthetics of the human body is a key feature that spectators appreciate in Slovak athletics. A bit smaller, but still high ratings were found in the domains of drama (Mdn = 6.2–6.7) and vicarious achievement (Mdn = 6.3–6.5). For comparison “vicarious achievement”, the feeling of success fans experience through a golfer's performance, is a primary driver in how spectators identify with the event and the sport (43).

Participants prefer the unpredictability of sports performance and success, with which they often identify. In the case of identification with success, the responses of indirect participants showed a predominance over those of direct participants, which means that indirect participants put themselves in the role of athletes and took their victories as their own. Strong identification with the team appears as an important factor that strengthens the attendees' positive attitude and their intention to attend a sporting event (44). The domains of aesthetics, escape, social interaction and acquisition of knowledge are also integral domains that contribute to the motivation of participants to attend the Slovak Athletics Championships. The lowest rated domain in both groups was acquisition of knowledge. This result indicates that direct and indirect participants do not come to the Slovak Athletics Championships mainly with the aim of learning something. For the Slovak Athletics Federation, this may mean other areas in which participants showed more positive responses.

We selected several relevant studies to make a comparison with our study. The first is a study conducted at the Spanish Athletics Championships, where the authors determined how factors such as staff, environment and sporting results influence attendees' overall experience and their willingness to return to the event. The study showed that sporting performance had the greatest impact on attendees' sense of fulfilment, and that women and attendees over 35 years of age perceived the quality of the event more positively than younger attendees (45). The study's conclusions confirm that understanding attendees' needs is key to achieving long-term sustainability of sporting events (45).

From the viewpoint of comparing the profile of participants in the Slovak Athletics Championships and the Spanish Athletics Championships, we found that the average age of participants was lower in our case. The average age of women in our study was 31.42 years, and in the Spanish study it was 36.00 years. The average age of men in our study was 31.00 years and in the Spanish study 33.00 years.

Measures following from research on motivational differences in participation in a sporting event recommend focusing on marketing, especially in the location where the sporting event will be held (17). It is also important to focus on the implementation of modern marketing strategies and building relations with the public (28). The level of services and equipment in the grounds significantly affects the satisfaction of spectators and their willingness to come to a sporting event (46). The authors of the study also confirmed this statement by finding that fans of a team whose infrastructure is of better quality are more motivated to come to an event (46).

The findings of the present study can be interpreted within the broader framework of spectator motivation and sport consumption theory. Previous research suggests that spectators attend sport events not only to observe the competition itself but also to experience entertainment, social interaction, and emotional engagement (13). In this context, the relatively negative evaluation of the accompanying program identified in the present study may indicate that the overall event experience does not sufficiently meet spectators' broader expectations beyond the athletic performance. Furthermore, studies on sport event satisfaction emphasize that the overall event environment, including atmosphere, entertainment elements, and spectator engagement activities, plays a crucial role in shaping spectator satisfaction and future attendance intentions (47). The results of this study therefore suggest that improving experiential aspects of athletics events may represent an important strategy for enhancing spectator engagement, particularly in smaller sports where media exposure and star power are more limited.

### Practical implications for the Slovak Athletics Federation

It is clear from the research results that the Slovak Athletics Federation should pay more attention to the needs of indirect participants in the Slovak Athletics Championships. The findings suggest that stronger promotional strategies and improved spectator experiences are key to attracting a wider audience and increasing the visibility of national athletics events in Slovakia (48). The absence of spectators without a previous connection to athletics points to a lack of effective audience development strategies. Improving the overall atmosphere of the event, adding accompanying event programmes and targeted promotion outside the athletics community may be key to building a sustainable spectator base and increasing the long-term visibility of national athletics events in Slovakia.

The results of the study indicate that the low attendance of the Slovak Athletics Championships is mainly influenced by poor interaction with spectators, low attractiveness of the programme and accompanying activities and weaker promotion of the event.

The findings also point out the fact that the current audience is mainly made up of athletes' family members and their friends. This problem is already of a long-term nature, but with appropriate and effective promotion of the event, this situation can be corrected. On the basis of this knowledge, we have formulated several practical recommendations for the Slovak Athletics Federation, which can be implemented and applied even with a limited marketing budget. Smaller-scale events, i.e., those that are not mainstream, naturally have a much harder time attracting spectators compared to top sports events, because they have less media reach and lower prestige (49).

One of the most effective strategies that the Slovak Athletics Federation can use in its promotion with a limited budget is interaction with spectators and fans through a campaign in which spectators and fans would send supportive videos or messages to their favourite athletes. The campaign would be called “Support Your Athlete”, and spectators and fans would send encouraging videos and messages addressed to specific athletes. These videos and messages would be projected on the screen during the competition itself and would serve as both interaction with spectators and also as motivation for athletes. When planning digital strategies, managers should take into account internal factors such as club tradition and culture, and external factors such as team performance and fan behavior (50). Competitions or raffles, in which prizes from partners and sponsors would be awarded, can also be a great attraction for direct spectators. This would increase the viewer experience and also effectively fulfil media obligations to partners and sponsors. In golf, marketing activities emphasize star athletes (43) and similar marketing activities, but in a limited form, are also used by the Slovak Athletic Federation.

The appropriate selection of digital communication tools also plays an important role in systematic communication, and not only with spectators and fans. One such tool is email marketing, which is suitable for regular communication with the athletic community. Communication through email marketing is effective and can help build awareness of events in Slovak athletics on a relatively low budget. In combination with paid promotion on the social media of the Slovak Athletics Federation, potential new spectators and fans can be reached even outside the narrow circle of the athletics community.

Strengthening work on social media through a person who would accompany viewers on social media can also bring positive benefits in terms of promotion. Such a person would be devoted to creating short videos, behind-the-scenes footage and interviews with athletes and coaches during the Slovak Athletics Championships; this would increase the online reach of the event and its attractiveness, especially for younger viewers and fans. To reduce costs, this work can be done by sports management students as part of their internship. In the case of a higher budget for promoting the event, one approach would be to contact sports influencers who would then be ambassadors of the event and help increase the visibility of the Slovak Athletics Championships.

We recommend that the Slovak Athletics Championships revive the cultural programme and create a fan zone that would provide viewers with an experience in the form of an autograph session, knowledge and movement competitions, kiosks, a photo wall and interactions with a mascot. We would also include performances by local dance groups and a singer who would sing the anthem live as a way to enhance the experience of the event. The quality of the services provided is equally important, especially refreshment options, which should better reflect the needs of athletes, coaches, family members and friends.

In the medium term, the association may consider developing additional tools to build fan loyalty and identity, such as a loyalty system based on collecting points for taking part in events or expanding the offer of official merchandise and its sale at events throughout Slovakia. These activities have the potential not only to strengthen the audience's relationship with athletics, but also to generate additional income.

Among the more financially demanding and long-term recommendations, we include the establishment of a separate marketing position at the Slovak Athletics Federation and the development of its own mobile application. As digital technology evolves, communication has shifted from a push model to a pull model, where users actively choose what information they access, when they access it, and how it is delivered (51). Carrying out these recommendations would significantly help increase attendance at athletics events in Slovakia.

The presented recommendations can also be applied to the Athletics League, which the Slovak Athletics Federation organises each year. The Athletics League is a team competition between clubs, in which athletes have the opportunity to collect points for the world rankings and compete with the best domestic athletes.

### Research limitations and future research directions

The originality of the study led to several limitations. In the study, we focused on one national athletics championship, which was held on 2–3 August 2025 in Banská Bystrica. Another limitation of the study was the low number of respondents (*n* = 39), which is due to the low attendance at Slovak Athletics Championships. In the future, we recommend comparing several national athletics championships, which would thus provide a lot of relevant data and allow us to generalise the results to a given location. By including a higher number of national athletics championships in a study, we could determine whether differences occur in terms of attendance of national athletics championships with regard to the venue, since there is only one athletics stadium in Slovakia that is suitable for organising international athletics events. It was at this stadium that the national athletics championships were held in 2025. The stadium is located in Banská Bystrica and the Summer European Youth Olympic Festival (2022) and the European Under-18 Championships (2024) have previously been organised there. Additional research in this area could focus on comparing several countries in terms of attendance at national athletics championships and also on comparing the Slovak Athletics Championships with other sports in which championships are held. Also limitation was that media analysis focused primarily on the quantity of promotional outputs rather than engagement metrics such as reach or interaction levels.

## Conclusion

The study provides a comprehensive look at the factors and areas that influence the low attendance at the Slovak Athletics Championships. The findings of this study are exploratory in nature, as they primarily reflect the perceptions of individuals already involved in athletics, either directly or indirectly, such as athletes, coaches, family members, and friends. In the analysis, we found that all participants had a direct personal or professional connection to athletics, and no independent spectators from the general public were identified in the sample. This indicates that the audience consisted exclusively of people from the athletics environment, not occasional or new attendees. Several factors influence the low attendance at the Slovak Athletics Championships, and these are caused by organisational shortcomings and the limited attractiveness of the event for the wider public, as well as its weak anchoring in the media and marketing space. This indicates that dissatisfaction among participants is widespread and the organisers should address the needs of all participants in the Slovak Athletics Championships. The main finding is that the Slovak Athletics Championships are organised primarily for athletes and coaches who are comfortable with the organisational setup. In contrast, family members and friends perceive the organisation, transportation and logistics negatively. This state indicates that the current organisational model underestimates spectator comfort in favour of the smooth running of the disciplines. The absence of an accompanying event programme was identified as the most significant barrier to building a spectator base. The high measure of dissatisfaction in this area confirms that without changes in this regard, it will not be possible to reach a wider audience outside the narrow circle of family members and friends. The research also showed that direct and indirect participants share a very similar psychological profile. Both groups are motivated mainly by the physical attractiveness of the athletes and the drama of the races. The identified similarity in motivations is a positive sign for the Slovak Athletics Federation that marketing communication can be targeted uniformly at both groups. We also presented recommendations that can be implemented even with a limited marketing budget for the event as a means of increasing the attractiveness of the event.

## Data Availability

The original contributions presented in the study are included in the article/Supplementary Material, further inquiries can be directed to the corresponding author.
